# Has the pelvic renal stone position inside the upper loop of JJ stent any influence on the extracorporeal shock wave lithotripsy results?

**DOI:** 10.1186/s40064-016-2954-2

**Published:** 2016-08-08

**Authors:** Catalin Pricop, Dragomir N. Serban, Ionela Lacramioara Serban, Alin-Adrian Cumpanas, Constantin-Virgil Gingu

**Affiliations:** 1 Department of Urology, “C. I. Parhon” Hospital, “Gr. T. Popa” University of Medicine and Pharmacy, Iasi, Romania; 2 Department of Physiology and Center for Study and Therapy of Pain, “Gr. T. Popa” University of Medicine and Pharmacy, Iasi, Romania; 3 Department of Urology, University Hospital, “Victor Babes” University of Medicine and Pharmacy, Timisoara, Romania; 4 Center of Urological Surgery and Renal Transplantation, Fundeni Clinical Institute, Bucharest, Romania

**Keywords:** Renal stones, Extracorporeal shock wave lithotripsy, Ureteral JJ stent

## Abstract

**Background:**

JJ stents are often encountered in patients with pelvic renal stones referred for shock wave lithotripsy, most of them being placed either for obstructive renal pelvic stones or for ureteric stones mobilized retrograde during the JJ stent insertion. The aim of the study was to determine whether the relative stone position in the upper loop of the JJ stent during extracorporeal shock wave lithotripsy (SWL) influences the efficiency of the procedure. The study was designed as a prospective cohort study on 162 patients addressing the same urological department, with single renal pelvic stone (primary or mobilized to the renal pelvis during the insertion of JJ stent), smaller than 15 mm, with JJ stent, treated by SWL using a second generation spark gap lithotripter, 18 kV, 3000 waves/session. Patients were divided in three groups according to the relative position of the stone to the upper loop of the JJ stent as appears on plain X-ray: stone-inside-loop, loop-crossing-stone and stone-outside the loop. The SWL success rate was the primary outcome of the study. p Value, Chi square and Kruskal–Wallis tests were used for statistical analysis.

**Results:**

For stone-inside-loop cases, SWL efficiency was 22.7 versus 42 % for all the other cases (p = 0.002). Other factors for decreased SWL success rate were: higher stone radio-opacity, larger JJ of stent and obese patients. Study limitation is represented by the relative small study group and by the evaluation of stone density using plain X-ray instead of computer tomography.

**Conclusions:**

For pelvic renal stones having the same density characteristics studied by plain X-ray, the SWL efficiency is lower in stone-inside-loop cases comparing with the other positions. The overall stone free rate for renal pelvic stones could be explained by the second generation lithotripter used for all procedures.

## Background

The treatment of urolithiasis by extracorporeal shock wave lithotripsy (SWL) is a relatively simple and accessible method, with an overall success rate between 60 and 90 % (Rao et al. 2011; Stoller and Meng 2007; Rassweiler et al. 2011; Pilar Laguna Pes et al. 2010; Tiselius 2009; Argyropoulos and Tolley 2007; Saigal et al. 2005; Krishnamurthy et al. 2005; Pareek et al. 2005; Seitz et al. 2006; Weld et al. 2007; Wiesenthal et al. 2010; Ouzaid et al. 2012; Alyami et al. 2012). When applied right after the renal colic onset, SWL is proved to be highly effective (Rassweiler et al. 2011; Pilar Laguna Pes et al. 2010; Tiselius 2009; Argyropoulos and Tolley 2007; Skolarikos et al. 2010). However, strategy optimization is still necessary. The placement of a JJ stent for recurrent colic and/or infected hydronephrosis often leads to retrograde stone mobilization to the intrarenal collecting system, further favoring SWL success rate, mainly due to the surrounding liquid that allows cavitation (Türk et al. 2014; Rassweiler et al. 2011; Tiselius 2009). The presence of a JJ stent can affect stone fragments elimination and there are data in literature suggesting that it can reduce SWL efficiency by directly perturbing shock waves (Tiselius 2009; Argyropoulos and Tolley 2009; Mohayuddin et al. 2009) as any structure dispersing the shock waves (e.g. foreign body, local edema) diminishes SWL success rate (Jain and Shah 2009). Stone density, size and composition are essential factors that influence SWL outcome (Türk et al. 2014; Rao et al. 2011; Stoller and Meng 2007; Rassweiler et al. 2011; Pilar Laguna Pes et al. 2010; Tiselius 2009; Argyropoulos and Tolley 2007; Saigal et al. 2005; Krishnamurthy et al. 2005; Pareek et al. 2005; Seitz et al. 2006; Weld et al. 2007). The shock waves transmission has a paramount importance for stone fragmentation (Williams et al. 2003). From the absence of the bubbles in the coupling medium, to the skin-to-stone distance and the stone density, all of these factors influence the physical processes of shock wave transmission and stone disintegration.

As the stent can interpose between the shock wave front and the stone, it can be hypothesized that this situation could impede the fragmentation process as well.

Computed tomography (CT) is appropriate to evaluate stone location, density and skin-to-stone distance, all predicting SWL success rate (6, 9–12), while the body mass index (BMI) is still a debated predictor (Seitz et al. 2006; Weld et al. 2007). However, despite its sensitivity and specificity, CT remains an expensive method. Due to the number of SWL procedures, the cost of CT procedures for the healthcare system could be significantly higher, while exposing the patient to a higher irradiation level compared with plain X-rays (Türk et al. 2014). Although CT remains the standard for estimation of stone density, the method has its limitations and there are authors suggesting plain X-ray has a good sensitivity and specificity and it can be used as a surrogate for CT (Lim et al. 2015; Motley et al. 2001).

The aim of our study was to evaluate whether the stone position relative to the JJ loop into the renal pelvis can influence the stone fragmentation process. If it is so, this factor-along high stone density and high BMI-could be taken into consideration for choosing the optimal treatment.

## Methods

This prospective study involved 162 consecutive adult patients with pelvic renal stones treated by SWL and fulfilled all the following inclusion criteria: single pelvic renal stone, <15 mm, visible on plain X-ray kidney–ureter–bladder (KUB), with JJ stent and without prior SWL treatment on the same side. Exclusion criteria were represented by the contraindications for SWL (pregnancy, coagulation disorders, aortic aneurysm and platelet aggregation inhibitors).

The pelvic renal stones were either primary located in the renal pelvis or secondary to retrograde stone mobilization from the ureter. The JJ stent was inserted for impacted pelvic renal stone or after the retrograde mobilization of the stone from the ureter. The inclusion period was June 2001–January 2015.

Before the insertion of JJ stent, the functional evaluation of the obstructed kidney for all patients was performed using intravenous urography (IVU).

**Fig. 1:**
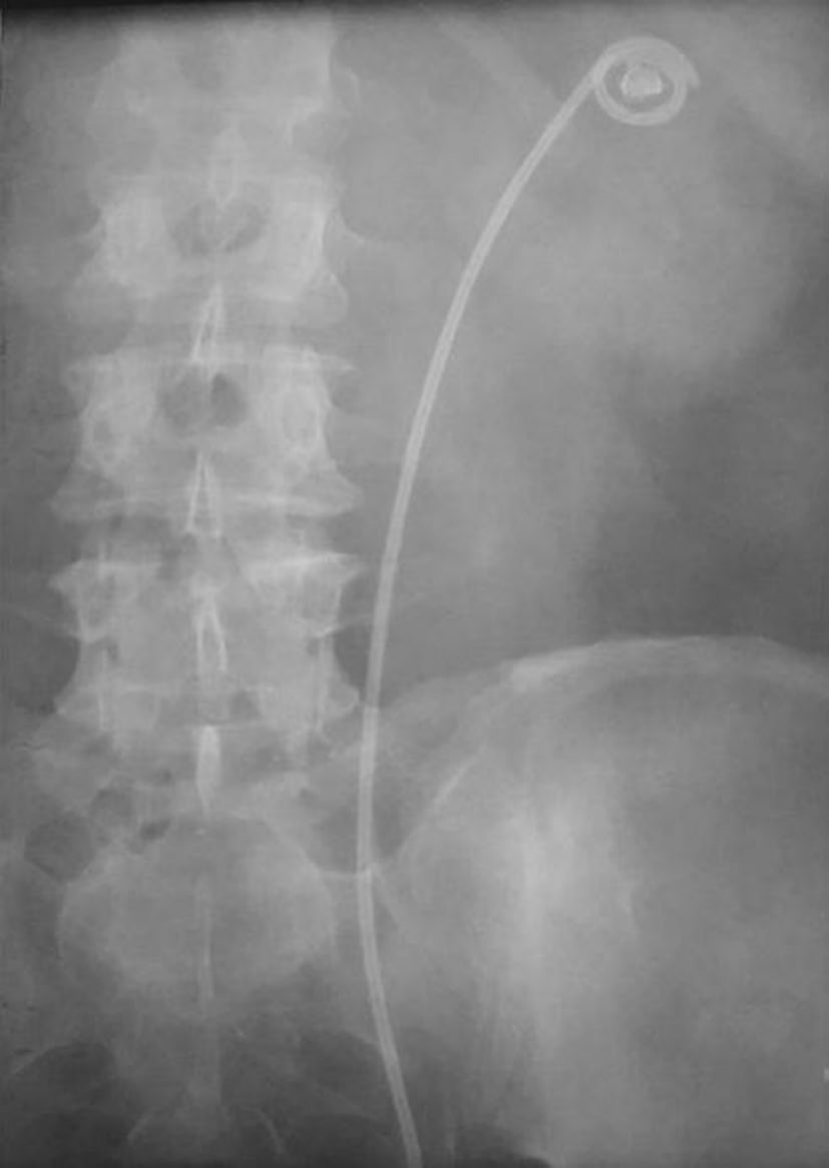
KUB of a patient from group A: stone inside the JJ loop

**Fig. 2:**
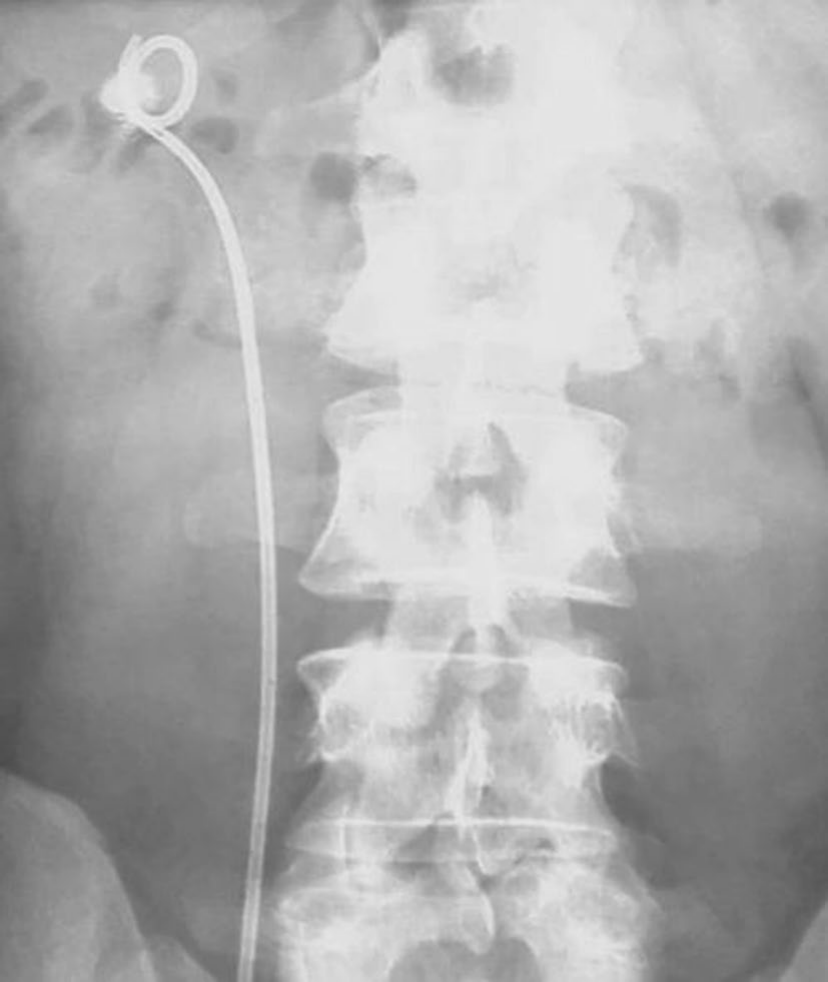
KUB of a patient from group B: loop-crossing-stone

**Fig. 3:**
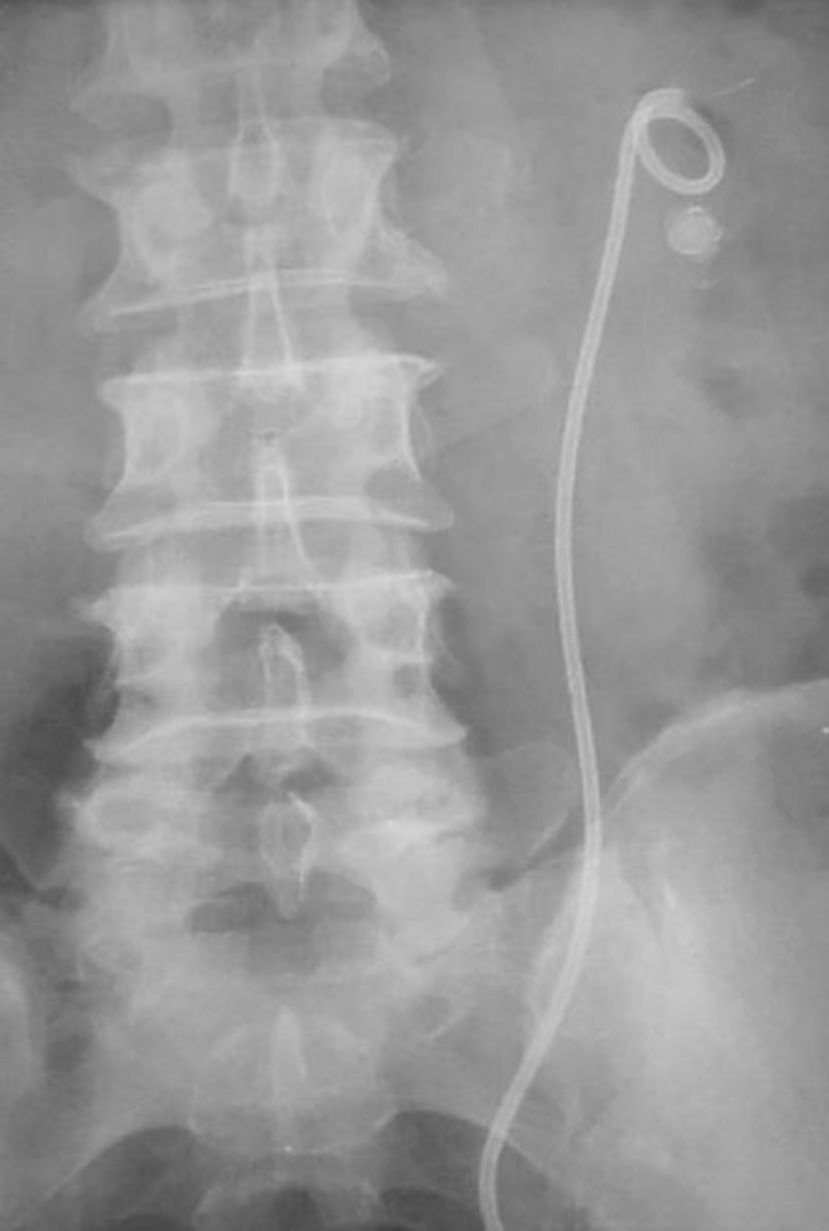
KUB of a patient from group C: stone outside the JJ loop

The patients were included in one of the three groups, based on KUB-defined relation between the stone and upper stent loop: group A—stone-inside-loop (Fig. 1), group B—loop-crossing-stone (Fig. 2) and group C—stone-outside-loop (Fig. 3).

The stone density, as revealed by KUB, was classified as intense radiopaque (IR—opacity similar to 12th rib or higher), moderate radiopaque (MR—opacity lower than the 12th rib), and slightly radiopaque (SR—stone barely visible). For preoperative assessment of the stone opacity, KUB was evaluated by two radiologists and in case of no similar result a third opinion was used to define the stone as IR, MR or SR.

The JJ stent (caliber Fr 6, Fr 7 or Fr 8) was previously inserted in each case only for emergency reasons: recurrent renal colic refractory to medical treatment or renal colic with obstructive stone and fever. All JJ stent insertions were uneventful, leading to stone migration to the renal pelvis. In case of urinary tract infection, antibiotics were prescribed prior to SWL. Only patients rendering sterile urine status were subsequently treated with SWL.

SWL was performed by the same urologist, having an experience of more than 1500 procedures, using a Chinese second generation spark gap lithotripter, model KS 88-4, with radiologic targeting system, 18 kV and a standard procedure of 3000 shock waves per session. A second and a third session were used as standard protocol at 3–4 weeks interval when the stone free status had not been rendered.

Patients from the study groups were followed-up monthly for at least 1 month. If they rendered the stone free status they would exit the study group. Patients who did not render the stone free status were treated in the second and, eventually, the third session. At 3 months after the 3rd SWL session, an alternative treatment was chosen. The follow-up of the patients included KUB and ultrasound (US) examination at 3–4 weeks post SWL. The results were defined as: stone-free (SF)—when no visible residual fragments were found on KUB and US, stone fragments (F)—when fragments of any dimension lower than the original stone size, including those smaller than 4 mm were found or stone not fragmented (NF)—when the stone size had the same size as before treatment. In order to avoid biases, postoperative evaluations were performed by the same experienced investigators. Two investigators were involved: one radiologist for KUB, blinded to ultrasound results and one urologist for ultrasound, blinded for KUB results. In case of lack of concordance between results, a case review and consensus meeting was imposed, by allowing both investigators to see both imagistic evaluations.

If stone free status was not obtained on KUB and ultrasound investigation, a second and if necessary a third SWL was performed.

A multivariate analysis regarding the influence upon SWL efficiency for stone radio-opacity (IR, MR or SR, respectively), stone size (<10 and 10–15 mm), BMI as defined by the World Health Organization (normal weight BMI 18.5–25, overweight BMI 25–30 and obese patients BMI >30) and JJ stent caliber (6 Fr, 7 Fr or 8 Fr) was realized.

JJ stents were retrieved after rendering the stone free status, no later than 3 months from insertion or upon request. In case of stent intolerance Tamsulosin 0.4 mg, once daily, was administered. After the completion of the study protocol, patients not rendering the stone free status were treated with PCNL, semirigid ureteroscopy for steinstrasse or by retrograde intrarenal surgery (RIRS).

The study design fulfilled all the legal and ethical requirements for IRB approval. All the patients signed the informed consent, according to the legal and ethical requirements, being involved in current use procedures, with no experimental aspect.

The results were processed and analyzed using the software package SPSS 17 (IBM Corporation). Data were categorized using unique identifiers and then processed to obtain the derived indicator, relative frequency of SWL outcome (SF, F, or NF) expressed as percentage of cases from the respective patient number. SWL outcome frequencies were tested statistically using the Chi square test, p < 0.05 being considered statistically significant. Finally, multivariate analysis was also applied, using the Kruskal–Wallis test.

## Results

The mean age of the patients was 48 ± 3 years (range 25–64), without statistical differences between the three groups. Male/female ration was 1.53. Out of 162 patients, sixteen had the stent removed upon request, out of the scheduled protocol although they did not reach the stone free status after one (n = 5), two (n = 6), or three sessions (n = 5), because it was hard to tolerate it despite Tamsulosin 0.4 mg/day along the whole period when they had the stent in place and Lornoxicam 8 mg/day, 10 days after each SWL. In order to avoid biases, these patients were excluded from the study. 12 cases with opinion differences regarding the results of SWL, as evaluated by KUB and ultrasound, were debated in order to achieve consensus.

Table 1 presents the other patient characteristics in the study groups, related to radio-opacity, BMI, stent caliber, and stone size. There were no statistically significant differences between the three groups regarding the above mentioned characteristics. In each of the three groups, the percentage of obese patients was insignificant.

**Table 1:** Patients’ characteristics within the study groups

	Group A		Group B		Group C	
	n	%	n	%	n	%
Total	44	100	34	100	84	100
*Stone radio*-*opacity*						
IR	15	34.1	12	35.3	29	34.5
MR	17	38.6	10	29.4	33	39.3
SR	12	27.3	12	35.3	22	26.2
*Stent caliber* (mm)						
8	12	27.3	8	23.5	14	16.7
7	10	22.7	3	8.8	10	11.9
6	22	50.0	23	67.6	60	71.4
*Body mass index (BMI)*						
OB (BMI >30)	3	6.8	0	0	3	3.6
OV (BMI 25–30)	17	38.6	11	32.4	22	26.2
N (BMI 18.5–25)	24	54.5	23	67.6	59	70.2
*Stone size* (mm)						
<10	23	52.3	15	44.1	35	41.7
10–15	21	47.7	19	55.9	49	58.3

*Group A* stone-inside-loop, *Group B* loop-crossing-stone, *Group C* stone-outside-loop, *IR* intense radiopaque (similar or superior to 12th rib opacity), *MR* moderate radiopaque (less opaque than 12th rib), *SR* slightly radiopaque (barely visible), *OB* obese, *OV* over-weight, *N* normal weight

 The results of SWL are presented in the left side of Tables 2, 3, 4 and 5, with the statistics for the relevant comparisons described in the right side of each table. The situation stone-inside-loop (group A) reduced SWL overall success rate, statistically significant comparing with group C (stone outside the loop) and with the overall results of group B and C altogether (p = 0.02 and p = 0.004 respectively). For stone-inside-loop cases SWL efficiency was 22.7 %, about twice lower than 49.1 % for the rest of cases (group B + C) (p = 0.002). SWL success rate was ~3 times lower for stone located in the JJ stent loop after two SWL sessions and remained ~2 times lower after the third session.

**Table 2:** The SWL outcome (case %) in the three groups and comparisons (Chi square p values) among the study groups

SWL outcome	Case %				*p* values				
	A	B	C	All	A versus B	A versus C	B versus C	A versus B + C	C versus A + B
SF after 1st SWL	4.5	2.9	11.9	8.0	0.819	0.300	0.243	0.503	0.110
SF after 2nd SWL	4.5	14.7	25.0	17.3	0.247	*0.009*	0.329	*0.017*	*0.013*
SF after 3rd SWL	13.7	23.5	15.5	16.7	0.406	0.987	0.441	0.693	0.833
Overall stone free	22.7	41.1	52.4	42.0	0.133	*0.002*	0.368	*0.004*	0.263
Residual fragments	36.4	32.4	27.4	30.9	0.897	0.397	0.752	0.463	0.409
Stone not fragmented	40.9	26.5	20.2	27.1	0.276	*0.022*	0.621	*0.028*	*0.040*

Values in italics for *p* < 0.05

*Group A* stone-inside-loop, *Group B* loop-crossing-stone, *Group C* stone-outside-loop, *All* the whole study group, *SF* stone-free

**Table 3:** The SWL outcome (case %) according to stone radio-opacity and comparisons (Chi square p values) among these groups

SWL outcome	Case %				p values				
	IR	MR	SR	All	IR versus MR	IR versus SR	IR versus other	SR versus MR	SR versus other
SF after 1st SWL	3.6	5.0	17.4	8.0	0.937	*0.045*	0.225	*0.040*	*0.015*
SF after 2nd SWL	10.7	16.7	26.1	17.3	0.510	*0.044*	0.165	0.345	0.102
SF after 3rd SWL	14.3	18.3	17.4	16.7	0.736	0.876	0.712	0.896	0.938
Overall stone free	28.6	40.0	60.9	42.0	0.272	*0.002*	*0.019*	*0.034*	*0.004*
Residual fragments	33.9	31.7	26.1	30.9	0.951	0.522	0.644	0.196	0.522
Stone not fragmented	37.5	28.3	13.0	27.1	0.394	*0.010*	*0.049*	0.098	*0.019*

Values in italics for *p* < 0.05

*IR* intense radiopaque (similar or superior to 12th rib opacity), *MR* moderate radiopaque (less opaque than 12th rib), *SR* slightly radiopaque (barely visible), *SF* stone free

**Table 4:** The SWL outcome (case %) according to body mass index (BMI) and comparisons (Chi square p values) among these groups

SWL outcome	Case %				p values				
	OB	OV	N	All	OB versus OV	OB versus N	OB versus OV + N	N versus OV	N versus OB + OV
SF after 1st SWL	0	0	12.3	8.0	–	0.797	0.977	*0.023*	*0.006*
SF after 2nd SWL	0	6.0	23.6	17.3	0.732	0.398	0.555	*0.014*	*0.007*
SF after 3rd SWL	0	2.0	24.5	16.7	0.200	0.375	0.577	*0.030*	*0.001*
Overall stone free	0	8.0	60.4	42.0	0.905	*0.013*	*0.035*	*0.001*	*0.001*
Residual fragments	0	32.0	32.1	30.9	0.246	0.228	0.223	0.862	0.779
Stone not fragmented	10	60.0	7.5	27.1	0.139	*0.001*	*0.001*	*0.001*	*0.001*

Values in italics for *p* < 0.05

*OB* obese (BMI >30), *OV* overweight (BMI 25–30), *N* normal weight (BMI 18.5–25), *SF* stone-free

**Table 5:** The SWL outcomes (case %) according to stent caliber and comparisons among these groups

SWL outcome	Case %				p values				
	8 Fr	7 Fr	6 Fr	All	8 versus 7 Fr	8 versus 6 Fr	8 versus 6 and 7 Fr	6 versus 7 Fr.	6 versus 7 and 8 Fr
SF after 1st SWL	2.9	8.7	9.5	8.0	0.726	0.384	0.383	0.786	0.515
SF after 2nd SWL	0	13.1	23.8	17.3	*0.032*	*0.004*	*0.006*	0.394	*0.006*
SF after 3rd SWL	2.9	4.3	23.8	16.7	0.652	*0.014*	*0.031*	*0.036*	*0.002*
Overall stone free	5.9	26.1	57.1	42.0	*0.033*	*0.001*	*0.001*	*0.014*	*0.001*
Residual fragments	23.5	47.8	29.6	30.9	0.105	0.648	0.102	0.148	0.747
Stone not fragmented	70.6	26.1	13.3	27.1	*0.002*	*0.001*	*0.001*	0.227	*0.001*

Values in italics for *p* < 0.05

*SF* stone-free

Confidence levels were particularly high (p < 0.01) for both the important comparisons directly relevant to the study main objective (A vs. C; A vs. B + C), regarding the endpoint frequency values for the stone-free status. We separately evaluated the influence of the other four factors: stone radio-opacity, stone size, BMI, and stent caliber. SWL efficiency generally depended upon stone radio-opacity (Table 3), BMI (Table 4) or stent caliber (Table 5) but was not influenced by stone size. On univariate analysis, the stone density, as evaluated by KUB, influences the stone free status when comparing intense with slightly radiopaque stones, from the first SWL session (p = 0.045) and this trend maintain to the second SWL session (p = 0.044). Overall stone free status is significantly lower on obese patients comparing the normal weight patients on a univariate analysis (p = 0.013). Meantime, the larger caliber of the ureteric JJ stent, the smaller the stone free status is, regardless of the location of the stone relative to JJ loop (Table 5).

The multivariate analysis combining all the above mentioned parameters using the Kruskal–Wallis test (Table 6) provided ultimate confirmation of the negative influence exerted upon SWL efficiency by the stone location inside the loop of the JJ stent, independent of the other factors.

**Table 6:** Multiple comparison regarding influences on SWL outcome in the studied patients; Kruskal–Wallis test with study group as the grouping variable

	Stone radio-opacity	Body mass index	Stent caliber	Stone size	Stone location
Chi square	0.241	3.503	5.169	1.319	13.007
*df*	2	2	2	2	2
Asymp. Sig.	0.886	0.174	0.075	0.517	*0.001*

Values in italics for *p* < 0.05

## Discussion

Although SWL is a safe procedure, with low risks, it is important to clearly define prognostic factors for the success and the failure. There are data in the literature suggesting that the presence of stents can decrease the efficiency of the SWL, none of the articles focusing on the relative stone position to the JJ loop (Türk et al. 2014). The hypothesis that the stent loop, interposing between the wave front and the stone, can influence the stone fragmentation has been verified by this study which, to our knowledge, is the first one addressing this issue. Interesting is that when comparing groups A and C, with the stone inside the loop and outside the loop, respectively, there is a statistically significant difference regarding the overall stone free status. The statistical significant difference in stone free status between the two groups manifests from the second SWL procedure for overall stone free status and for complete non-fragmentation. In other words, a patient with a stone inside the loop has a higher probability to a complete no-fragmentation of the stone, leading to another therapeutic option (Table 2). The intermediate position of the stone (group B), offers borderline results, probably influenced by the stone exposure to the shock waves.

One can observe that the overall stone free rate is lower than reported in the literature. Probably this is a result the individual performance of the second generation spark lithotripter model used in all of our procedures (Elkoushy et al. 2011).

Another interesting aspect to discuss is the role of KUB as a surrogate for CT to evaluate the stone density. As the stone attenuation on radiologic examination reflects the stone density, the two methods can be used for assessing the stone density (Motley et al. 2001). In our study, all the cases were evaluated by KUB due to the lack of the CT availability in our area during the study period. However, there could be two other reasons for choosing plain X-ray for estimating the stone density: the first one is related to the costs, which are significantly lower than for CT and this could be an important aspect for the emerging economy countries. In these countries the plain X-ray is often more accessible than CT. The other reason could be the irradiation exposure during a CT procedure which is 4–5 times higher for CT in comparison with plain X-ray (Türk et al. 2014). The development of multi-detector row CT devices as well as new protocols for low-dose (<3 mSv for the entire examination) and ultra low-dose CT (0.4–0.6 mSv) examinations in urolithiasis allows to reduce the radiation exposure up to 50 and 95 % respectively, compared with standard-dose CT (Sung et al. 2011; Kluner et al. 2006). Facilitated by the high contrast between the stone and the adjacent soft tissue, these new methods avoid excessive irradiation of the patient, having the sensitivity and specificity comparable with the standard CT and the radiation exposure comparable with KUB. Acknowledging the limitations of KUB in evaluation of stone density, it should be mentioned that there are published data which support its role as surrogate for CT (Lim et al. 2015; Bon et al. 1996; Dretler 1988; Bradley and Rao 2011).

The stone radio-opacity, related to stone composition affects SWL outcomes (Pareek et al. 2005; Argyropoulos and Tolley 2009; Mohayuddin et al. 2009) and our results confirm this data. Interestingly, our data suggests that there is a statistically significant difference between IR and SR for the first and the second SWL session but it is not the case of the 3rd SWL session. This can be explained by the possibility of having cystine stones or struvite stones, slightly radiopaque but poorly responsible to SWL. Stone size is known to influence SWL success (Rassweiler et al. 2011; Pilar Laguna Pes et al. 2010; Tiselius 2009; Argyropoulos and Tolley 2007; Seitz et al. 2006), but in our study there are not significant differences, mainly due to the stone size, up to 15 mm, and to the stone position into the renal pelvis.

The role of JJ stenting in renal colic is debatable. As immediate SWL in acute colic is safe and efficient (Rassweiler et al. 2011; Pilar Laguna Pes et al. 2010), a JJ stent in proximity of an ureteric stone does not help SWL and might even hamper fragment elimination despite fluid presence following the relief of obstruction (Argyropoulos and Tolley 2009; Mohayuddin et al. 2009). The indication for ureteric stenting in renal colic remains limited to untreatable pain and/or associated to urinary tract infection. In this last situation, the SWL is prohibited until the urinary tract infection is treated (Türk et al. 2014). Retrograde mobilization of the ureteric stone into the renal pelvis barely improves SWL efficiency (Bradley and Rao 2011). Thus, an endoscopic approach aiming to mobilize the stone, besides insertion of the JJ stent to relief an obstructed kidney is widely abandoned (Shen et al. 2011; Musa 2008).

In case of SWL on stented versus non-stented patients, including patients with single renal pelvic stone up to 20 mm, similar success rates were noted, with less renal colic but similar fever frequencies, similar or reduced “steinstrasse” frequencies, and higher frequency of low urinary tract symptoms (LUTS), all with higher cost (Argyropoulos and Tolley 2009; Mohayuddin et al. 2009; Cass 1992; Shen et al. 2011). None of these studies addressed the effect of stone position relative to the stent loop. Our results suggests that for a stone inside the JJ loop, the stone free rate decreases significantly as the JJ stent caliber increases from 6 to 8 Fr, probably by increasing the shield-like effect against the shock-wave front. From here can emerge the idea that a smaller caliber of the JJ stent could be a prevention measure for reducing the disadvantage of stone position inside the stent loop.

SWL efficiency is lower in obese patients (Stoller and Meng 2007; Pilar Laguna Pes et al. 2010; Argyropoulos and Tolley 2007; Weld et al. 2007; Wiesenthal et al. 2010; Ouzaid et al. 2012; Alyami et al. 2012) and our study supports the observation of the influence of BMI on SWL outcome in patients with JJ stent (Table 4). SWL outcome improves if the stone is in the intrarenal collecting system (well surrounded by liquid) and BMI is a fairly good indicator of success, besides actual skin-to-stone distance and stone radio opacity (Seitz et al. 2006; Weld et al. 2007). Shock waves space–time distribution with homogenous pressure on the stone would allow high efficiency of disintegration mechanisms, so older devices should be at least as efficient as newer ones (Rassweiler et al. 2011; Pilar Laguna Pes et al. 2010). With a third generation lithotripter the only prognostic factors were stone size and the presence of the stent, neither stone localization nor the BMI, probably due to a better shock wave penetration (Hatiboglu et al. 2011). With Dornier HM3 used for ureteric stones >10 mm, BMI independently predicted SWL success rate and for IR renal stones prognosis was determined by age, BMI and stone number. Stone burden, single stone and renal pelvis location were found to be the most favorable prognostic factors (Hatiboglu et al. 2011). Such studies also confirmed decreased SWL success in the presence of a JJ stent (Shen et al. 2011; Musa 2008), but none has evaluated the relation between the stone position and the JJ loop.

The study limitations are represented by the size of the study group and by the use of plain X-ray as surrogate for CT in estimation of stone density, the advantages and disadvantages being discussed above. A further larger study is ongoing, trying to avoid the current study limitations.

The data from our study could lead to practical considerations as follows: a patient with a renal pelvic stone inside the stent loop would have a small chance to render the stone free status after two SWL sessions comparing with one having the stone outside the loop. Obesity, intense stone radio opacity and a JJ stent caliber more than 6 Fr would further decrease the stone free rate probability. In these situations, taking into consideration the discomfort produced by the stent and the risk of upper urinary tract infection due to the presence of JJ stent, more invasive methods as RIRS or percutaneous nephrolithotomy should be taken into consideration. This approach could avoid patient discomfort and risks, as well as money and time waist.

## Conclusions

Our results seem to sustain the hypothesis that stone-inside-loop relation reduces the SWL efficiency as an independent parameter on a multivariate analysis. In patients with single renal pelvic stone and JJ stent, the stone-inside-loop position lowers SWL success. In correlation with other prognostic factors, the relative position of the stone to the JJ loop could be a helpful tool to choose the most appropriate treatment for the patient, minimizing the discomfort and the costs. The overall stone free rate—lower than the results published in the literature for the renal pelvic stones—could be explained by the second generation lithotripter we used for all procedures.
